# An Avalanche of Modern Drugs

**Published:** 1895-11

**Authors:** 


### An Avalanche of Modern Drugs.

The *materia medica* of the old-time physician was of the simplest. The few drugs that he carried with him in his saddle-bags served him, either singly or in combination, for a great variety of purposes, and with them he treated—and in no small proportion of cases cured—all the common ills that flesh is heir to. He would have stared with astonishment at the avalanche of chemicals that come tumbling from the manufactories by dozens nowadays, each possessing therapeutic properties more or less accurately ascertained. Of this modern embarrassment of medical riches *The Hospital* speaks in a leading article bearing the suggestive title "Drugs Many; Remedies Few." We quote a few passages for the benefit of our readers:

"'New drugs are added every day for the benefit chiefly of those who do not know how to employ the old ones.' Such is the verdict of Sir William Broadbent, pronounced in the presence of his professional brethren assembled in annual congress under the auspices of the British Medical Association. By way of set-off to this may be placed the opinion of the average patient, who plumes himself upon his family physician or favorite consultant because the learned gentleman is so thoroughly 'up-to-date,' and so prompt in the application of 'all the new things.'"

After reminding us that the origin of most of the new remedies is in the enterprise of the pharmaceutical chemist, and disclaiming any intention of belittling this enterprise, since its rise is "a development for which medicine must be grateful," the article goes on to say:

"But there is another side to the question. * * * What, then, in a word, is the point of Sir William Broadbent's judgment? It is this, that many men never set themselves to prove experimentally for themselves the value of any drug or drugs, and so they never come to a condition of mind in which they employ remedies with confidence, precision, and success. The story of King David's sling and stone has a priceless application in medicine. It is infinitely better for a man to confine his practise to the use of a dozen drugs of which he has long and intelligently studied the various applications, than to employ a thousand of most of which his knowledge is merely guess-work. * * *

"It is the ruin of modern medicine that men do not use heir minds and base their work on the immovable foundation of their own proved convictions. They are not even so steady as kites in the air, which are at least moored to the earth by strings. Their counterpart is the thistle-down; and they are carried here, there, and everywhere, nobody knows where, by every therapeutic puff of wind that blows.

"Is there, then, no place for new remedies and new methods in modern medical practice? There is room of the amplest; room and to spare. What we insist upon is this twofold principle, that the old which is tired and proved shall be loyally preserved, that well-known drugs shall be retained, and that the new shall always, in every case, and by every individual, be subjected to continuous and competent examination and proving. This, we say, is not recondite philosophy; it is the most elementary common sense. But it is for the lack of this elementary common sense in daily practise that thousands of practitioners are soldiers bearing rifles but who can not shoot, sailors in commend who can not navigate their own ships. The third-rate practitioner, who has not gained a just self-confidence by reason of thoroughness and success in practice, always hankers after the reputation of being thoroughly 'up-to-date.' The British Medical Association will not have meet in London in vain if all men of this order, and all their patients also, will seriously lay to heart Sir William Broadbent's words, that 'new drugs are added every day for the benefit chiefly of those who do not know how to employ the old ones.'"

